# Optimized protocols for generating half-sized embryos from separated first two blastomeres in green sea urchin and *Xenopus laevis*

**DOI:** 10.3389/fcell.2025.1730288

**Published:** 2025-12-18

**Authors:** Eugeny E. Orlov, Polina S. Timoshina, Elena A. Parshina, Fedor M. Eroshkin, Maria A. Bannikova, Andrey G. Zaraisky

**Affiliations:** 1 Shemyakin-Ovchinnikov Institute of Bioorganic Chemistry, Russian Academy of Sciences, Moscow, Russia; 2 Koltzov Institute of Developmental Biology of Russian Academy of Sciences, Moscow, Russia

**Keywords:** *Xenopus*, sea urchin, blastomere separation, twins, embryonic scaling

## Abstract

The ability to restore normal body proportions after size reduction is a remarkable feature of early development. At the dawn of experimental embryology, Hans Driesch demonstrated that separated sea urchin blastomeres can develop into fully proportioned organisms, revealing an intrinsic capacity for embryonic scaling. However, the molecular mechanisms that enable this phenomenon remain poorly understood. Modern investigations of scaling, particularly those relying on bulk omics approaches, require reliable methods for producing large numbers of embryos that differ in size from wild-type embryos. Here, we present optimized protocols for generating half-sized embryos from separated blastomeres in two phylogenetically distant model organisms: the green sea urchin (*Strongylocentrotus droebachiensis*) and the frog (*Xenopus laevis*). These updated methods build on classical embryological approaches and enable robust, reproducible production of half-sized embryos. The resulting embryos are well suited for downstream applications, including *in situ* hybridization, morphogen gradient analysis, and high-throughput molecular profiling such as RNA sequencing and proteomics. Together, these protocols offer a powerful platform for investigating the genetic and physical principles that govern embryonic scaling across diverse deuterostome lineages.

## 1 Introduction

A fundamental property of embryos in many species is their ability to proportionally adapt body structure to changes in overall size. While adult organisms typically rely on regeneration to restore lost tissues, early embryos can adjust their developmental patterning to altered size with striking precision, a phenomenon known as embryonic scaling. This capability was first described by Driesch (1891), who observed that separated blastomeres of sea urchin embryos could give rise to complete, proportionate larvae. Similar findings were reported soon after by Morgan (1895) in amphibian embryos. Since then, embryonic scaling has been documented in a wide range of animals, including cnidarians (Fritzenwanker et al., 2007), arthropods (Holm, 1952; Sander, 1971), fish (Hoadley, 1928; Almuedo-Castillo et al., 2018), amphibians (Ben-Zvi et al., 2008; Inomata et al., 2013), birds (Khaner, 1996; Caldarelli et al., 2024) and mammals (Katayama et al., 2010).

This process is thought to depend on organizer regions that produce long-range morphogen gradients. In amphibians and other models, such gradients are established by secreted signals originating from territories like the Spemann–Mangold organizer (Spemann and Mangold, 1924; Reversade and De Robertis, 2005; Lee et al., 2006), or by analogous organizers in other species (e.g., Holm, 1952). Yet, the precise molecular mechanisms by which these gradients adjust to embryo size remained unclear for decades.

Advances in developmental theory over the past century have provided a framework for understanding embryonic scaling in terms of dissipative self-organization and reaction–diffusion (RD) systems. In these systems, morphogens with different diffusion and degradation rates form spatial gradients that direct tissue patterning (Turing, 1952; Gierer and Meinhardt, 1972; Meinhardt, 1982; Čapek and Müller, 2019). Experimental work has shown that feedback regulation and diffusible modulators can alter these systems, enabling dynamic scaling of patterning fields (Ben-Zvi et al., 2008; Almuedo-Castillo et al., 2018; Inomata et al., 2013; Caldarelli et al., 2024).

In particular, to systematically investigate the molecular regulation of scaling, we have proposed the Scalers Hypothesis, which posits that all scaling-competent patterning systems must include size-sensitive components called scalers, whose concentrations vary with embryo size and influence morphogen gradient dynamics (Nesterenko and Zaraisky, 2019; Orlov et al., 2022; Timoshina et al., 2024). These scalers function both as sensors of embryo size and as modulators of gradient formation. They are encoded by specific genes whose expression levels respond to changes in embryonic size. Using this conceptual framework, we identified Matrix metalloproteinase 3 (Mmp3) as a key scaler of BMP/Chordin/Noggin gradients in *Xenopus laevis*, and later BP10 and SpAN as analogous scalers in the green sea urchin *Strongylocentrotus droebachiensis*. These genes exhibit size-dependent expression and encode proteases that regulate Chordin stability, enabling precise modulation of morphogen gradients and promoting robust scaling of embryonic patterning.

To experimentally investigate embryonic scaling using omics approaches such as RNA sequencing, proteomics, and related methods, it is essential to generate large numbers of embryos that differ in size from wild-type embryos. In this work, we present optimized protocols for producing half-sized embryos through blastomere separation at the two-cell stage in *Xenopus laevis* and the green sea urchin. These procedures build upon and refine earlier methods described for *Xenopus laevis* by Cooke and Webber (1985a), Cooke and Webber (1985b), and Kageura and Yamana (1983), as well as protocols developed for sea urchin embryos by Cameron et al. (1996) and Ortiz et al. (2019).

For sea urchins, our protocol enables the production of thousands of half-sized embryos suitable for bulk RNA-seq, SDS-PAGE, immunostaining, or morphogen quantification. Although originally developed for the cold-water species *Strongylocentrotus droebachiensis*, the method is readily adaptable to the widely used *Strongylocentrotus purpuratus*.

These updated protocols enable rigorous comparative analyses of size-dependent gene expression and morphogen behavior, facilitating broader investigations into the molecular logic of embryonic scaling.

## 2 Materials and equipment

### 2.1 Reagents and solutions for the *Xenopus* protocol

- 0.1× MMR (Marc’s Modified Ringer’s solution, pH 7.4). Prepared as described in Shitikov et al. (2024).
- 0.6× MMR (pH 6.4). To prepare this solution:
  ○ First, generate 1 M stock solutions of Na_2_HPO_4_ and NaH_2_PO_4_.
  ○ Mix the phosphate buffers in a ratio of 26.5:73.5 (Na_2_HPO_4_:NaH_2_PO_4_) to produce 1 M PBS at pH 6.4.
  ○ Add this PBS to 0.6× MMR (prepared without HEPES) to achieve a final phosphate concentration of 20 mM.

### 2.2 Equipment for the *Xenopus* protocol

The following instruments and materials were used during *Xenopus* blastomere separation and downstream handling:

- Leica KL300 LED dissecting microscope
- Leica M205 stereomicroscope with Leica DC400F camera
- Dumont jeweler’s fine forceps
- Glass capillaries with 1 mm and 1.2 mm outer diameters (for well formation and microinjections)
- Bunsen burner
- 100 mm plastic Petri dishes
- Plastic Pasteur pipettes

### 2.3 Reagents and solutions for the *Strongylocentrotus droebachiensis* protocol

- Seawater. *S*. *droebachiensis* embryos should be cultured in chilled, filtered natural seawater or artificial seawater of matching salinity.
- 3-Amino-1,2,4-triazole stock solution (1 M, 1,000×). Dissolve 0.84 g of 3-amino-1,2,4-triazole in 10 mL of distilled water. Store at 4 °C.
- Calcium- and magnesium-free seawater (CMFSW). To prepare 1 L of CMFSW, combine the following:
  ○ NaCl: 26.24 g
  ○ KCl: 0.671 g
  ○ Na_2_SO_4_: 4.687 g
  ○ NaHCO_3_: 0.18 g
  Then add 10 mL of 0.25 M EGTA (pH 8.0) to the final solution.
  Adjust the pH if necessary. Prepare fresh or store at 4 °C.

### 2.4 Equipment for the *S. droebachiensis* protocol

The following equipment was used for blastomere separation and manipulation of *S. droebachiensis* embryos:

- Leica KL300 LED dissecting microscope
- 10 mL syringe fitted with a 23G × 1″ needle (for gentle embryo separation)
- Glass beakers (for embryo collection and rinsing)
- 100 mm glass Petri dishes
- Cold packs (to maintain seawater temperature during handling)
- Pipette or pipette controller fitted with a wide-bore glass capillary

## 3 Methods

### 3.1 Protocol for generating half-sized embryos from separated first two blastomeres in *Xenopus laevis*

This procedure is adapted from established methods (Kageura and Yamana, 1983; Cooke J. and Webber J. A., 1985; Cooke J. and Webber, J. 1985). Standard protocols for oocyte collection, *in vitro* fertilization, and early embryo culture are described in detail by Martynova et al. (2021). Method of whole-mount *in situ* hybridization are available in Orlov et al. (2022).

#### 3.1.1 Preparation of agarose-coated petri dishes

To support normal development of separated blastomeres, embryos must be cultured in rounded agarose wells that mimic the spatial constraints of the vitelline membrane. Placement on flat surfaces results in cleavage along a single horizontal plane and typically leads to abnormal development or lethality.

The preparation steps are as follows:

1. Melt 2% agarose (electrophoresis-grade) in 0.6× MMR (pH 6.4) and pour the solution into a 100 mm plastic Petri dish to a depth of approximately 5 mm. Allow to solidify.
2. Seal the tips of 1 mm and 1.2 mm glass capillaries using a Bunsen burner until a rounded end is formed (Supplementary Figure S1A):

1.0 mm tips are suitable for most half-sized embryos.

1.2 mm tips may require additional heating to form wider wells for larger embryos.

**FIGURE 1:**
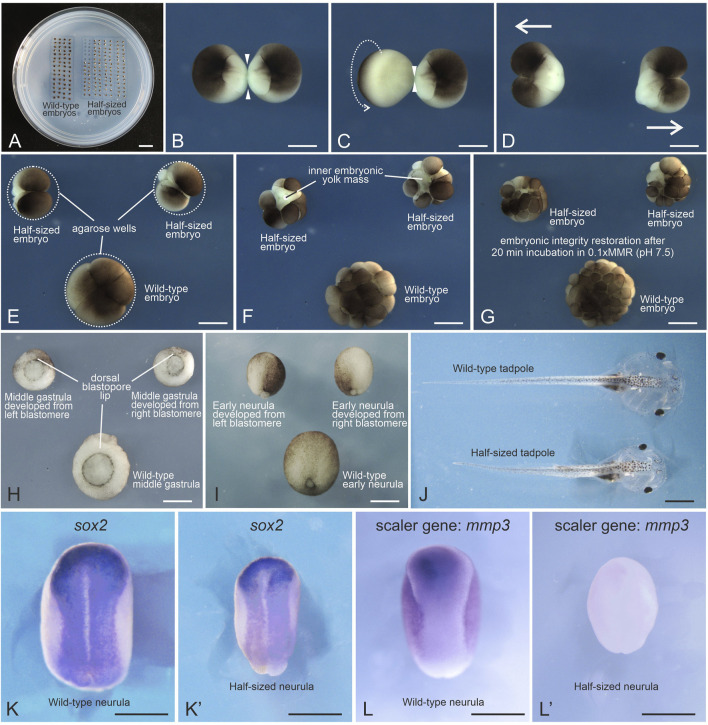
Generation of *Xenopus laevis* half-sized embryos. **(A)** Petri dish with an agarose bed containing rows of wells with wild-type and half-sized embryos. **(B)** Devitellinized embryo at the first cleavage stage forming a narrow isthmus (arrowheads) between the two blastomeres. **(C)** Arrow indicates the direction of rotation applied with forceps to stretch the isthmus into a thin, thread-like bridge. **(D)** Blastomeres immediately after separation. **(E)** Separated blastomeres placed in agarose wells initiate the second cleavage. Their wild-type sibling in the bottom well is at the same stage. **(F)** Some half-sized embryos exhibit instability and transient surface damage due to an increased surface-to-volume ratio. **(G)** Lowering ionic strength promotes re-adhesion of dissociated blastomeres within 10–60 min **(H,I)** Half-sized embryos at mid-gastrula and early neurula stages, alongside their wild-type siblings. **(J)** Half-sized and wild-type tadpoles at stage 46. **(K,K′)** *In situ* hybridization using a digoxigenin-labeled probe for the neural plate marker *sox2* mRNA reveals perfect scaling of the neural plate in the half-sized embryo at the mid-neurula stage. **(L,L′)** *In situ* hybridization using a digoxigenin-labeled probe for the scaler gene *mmp3* mRNA shows a marked decrease in expression in the half-sized embryo compared to its wild-type sibling at the mid-neurula stage. Scale bars: **(A)** 10 mm; **(B–L′)** 500 μm.

1. Reheat the tip and gently press it into the agarose surface to create wells. Approximately 70 paired wells can be prepared per dish to accommodate one experiment with both experimental and control embryos (Supplementary Figure S1B and Figure 1A).
2. Store the prepared dishes at room temperature in a sealed container to prevent dehydration until use.

#### 3.1.2 Separation and further culturing of the first two blastomeres

Egg collection, fertilization, and jelly coat removal were performed using standard protocols (Martynova et al., 2021), with the following modifications: (1) dejellying was initiated approximately 40 min after fertilization; and (2) embryos were incubated in cysteine solution for at least 10 min.

1. Wash the embryos five times in 0.1× MMR (pH 7.4) for 1 min each.

*Note:* At this stage, one-cell embryos may be transferred into 4% Ficoll in 0.1× MMR (pH 7.4) for microinjection. Select only healthy, fertilized embryos, i.e., those that do not adhere to the dish, have a glossy vitelline membrane, and exhibit a pigment stripe (indicative of cortical rotation). Avoid injecting precisely at the animal pole center; lateral injections are more likely to preserve cleavage symmetry. After injection, proceed directly to Step 2.

2. Prepare 4% Ficoll in 0.6× MMR (pH 6.4). After the final wash, replace the medium with this Ficoll-containing solution.

*Note:* 0.6× MMR at pH 6.4 can be substituted with a HEPES-buffered version at pH 7.4. However, better survival of half-sized embryos was observed in the pH 6.4 condition.

3. Transfer embryos to agarose-well Petri dishes pre-filled with 0.6× MMR (pH 6.4). Remove vitelline membranes with forceps and position the embryos with their animal poles facing upward.

*Note:* The cleavage plane is not predictable at the one-cell stage, requiring additional monitoring. An alternative is to wait for cleavage initiation and select symmetrically dividing embryos, but this narrows the time window for separation. Both pre-cleavage and early cleavage-stage strategies are viable and yield comparable results.

4. Observe first cleavage: Devitellinized embryos begin forming a dumbbell shape as the cleavage progresses and the isthmus narrows (Figure 1B).

*Note*: Do not prolong pre-cleavage incubation at low temperatures. It can impair isthmus thinning, possibly by affecting actomyosin activity (von Dassow et al., 2014). Maintain embryos at 20 °C–22 °C. Brief (5–10 min) exposure to colder temperatures is acceptable.

5. Rotate one blastomere gently against the other 2–3 times to stretch the isthmus into a thin thread-like bridge (Figure 1C). Wait ∼1 min to allow stabilization.
6. Separate the blastomeres by gently aspirating one of them with a plastic Pasteur pipette (Figure 1D).

*Note*: Avoid mechanical separation with forceps as it increases the risk of damage.

7. Place separated blastomeres into individual agarose wells with the animal pole facing upward (Figure 1E).
8. Devitellinize control embryos and transfer them into larger wells in the same Petri dish (Figure 1E).
9. Incubate embryos in 0.6× MMR (pH 6.4) until control embryos reach the 16–32-cell stage. Half-sized embryos may exhibit instability and transient surface damage due to their increased surface-to-volume ratio (Figure 1F).
10. Replace the medium by aspirating the 0.6× MMR and adding 0.1× MMR (pH 7.4). Perform two more washes with fresh 0.1× MMR.

*Note:* Lower ionic strength facilitates re-adhesion of dissociated blastomeres. Reconnection is typically observed within 10–60 min (Figure 1G).

11. To reach gastrulation, incubate overnight at 15 °C (Figure 1H).

*Note:* Avoid extreme incubation temperatures. Half-sized embryos are more vulnerable to thermal stress and may disaggregate at 12 °C or 25 °C.

12. Select viable half-sized embryos for downstream analyses, including further development into tadpoles or whole-mount *in situ* hybridization (Figures 1I–L’).

*Note:* To extend the time window during which blastomeres can be separated with minimal damage, that is from the onset of cleavage until re-adhesion, briefly transfer embryos to 12 °C just before cleavage begins. This cooling step slows development and provides more flexibility during manipulation. Then process embryos in batches of 15–20 for devitellinization at room temperature (20 °C), since lower temperatures may lead to isthmus thickening in some embryos. Depending on experience, approximately 10–20 embryos can be separated into individual blastomeres within 30 min.

### 3.2 Protocol for generating half-sized embryos from separated first two blastomeres in *Strongylocentrotus droebachiensis*

This protocol describes a high-throughput method for generating half-sized sea urchin embryos by separating blastomeres at the two-cell stage. It is based on a previously established method developed for relatively warm-water species *Strongylocentrotus purpuratus* (Cameron et al., 1996) and *Paracentrotus lividus* (Ortiz et al., 2019), which uses mechanical aspiration in calcium-free seawater, and has been adapted here for the cold-water species *S. droebachiensis* and for large-scale embryo handling.

#### 3.2.1 Temperature regime

*Strongylocentrotus droebachiensis* is a cold-water species native to boreal marine environments. Data from literature report, that at least adults can tolerate a wide range of temperatures: from −1.9 °C to 22 °C (review in Siikavuopio et al., 2012). Normal early development require low temperatures, such as 3 °C (Bögner et al., 2014). Stephens (1972) observed successful development at 0, 4 °C and 8 °C and reported that temperatures above 10 °C cause gross asynchrony in cell division, while 14 °C–15 °C were lethal. Due to the wide habitat, we suppose that there are population-specific differences in temperature tolerance. In our study, adults were collected from the Barents Sea near Murmansk (Russia) and transported to the laboratory by air. Embryos were cultured at 3 °C–4 °C, although development at +8 °C was also successful but more abnormalities were observed.

To minimize temperature-related defects, all seawater and glassware should be pre-chilled. When possible, all manipulations should be conducted in a cold room. In our setup, a standard refrigerator was used for embryo incubation, while cold packs were placed beneath Petri dishes to maintain low temperatures during manipulation. Glass dishes were preferred over plastic due to better thermal retention. Embryos were kept outside the refrigerator for as short a time as possible.

#### 3.2.2 Separation and further culturing of the first two blastomeres

Gamete collection and fertilization were performed according to standard protocols (Stepicheva and Song, 2014; Yaguchi, 2019; Timoshina et al., 2024). The jelly coat was not removed; instead, eggs were filtered through a coarse mesh (pore size >150 μm) to eliminate debris and spines. Fertilization was carried out in seawater supplemented with 1 mM 3-amino-1,2,4-triazole to inhibit hardening of the fertilization envelope.

1. Wash the zygotes twice in clean seawater and distribute them into 100 mm glass Petri dishes or 200–250 mL beakers.
2. Allow embryos to progress to the two-cell stage.

*Note*: Timing depends on temperature. At 8 °C, the first cleavage occurred at ∼3.5 h post-fertilization (hpf), while at 4 °C it was delayed to ∼5–5.5 hpf, which corresponds to data from literature (Stephens, 1972).

3. Remove a small number of two-cell embryos to a separate Petri dish to serve as stage-matched controls. These are essential for accurate staging and reduce the need to handle fragile experimental embryos.
4. Transfer the remaining embryos to a clean beaker. Remove excess seawater and wash the embryos twice with calcium- and magnesium-free seawater (CMFSW), allowing a total incubation time of ∼15 min. While embryos settle, prepare Petri dishes with standard seawater for post-separation culture.
5. Aspirate the embryos into a 10 mL syringe (without needle). Attach a 21G × 1.5″ needle and gently expel the embryos into the prepared Petri dishes using moderate pressure.

*Note:* Gentle aspiration may not separate blastomeres; excessive force may damage them. Monitor the separation process under a dissecting microscope.

*Additional notes:* Once separated, the blastomeres lose their fertilization envelopes and tend to become adhesive, forming clumps. To mitigate this:

○ Avoid overcrowding in dishes.
○ Minimize dish movement until embryos reach the swimming blastula stage.

Control embryos remain enclosed and do not clump, making them useful for developmental staging. In our experience, embryos did not adhere to untreated glass, but if adhesion becomes a problem, dishes may be coated with 1.5% agarose (Satoh and Kominami, 2008).

**FIGURE 2:**
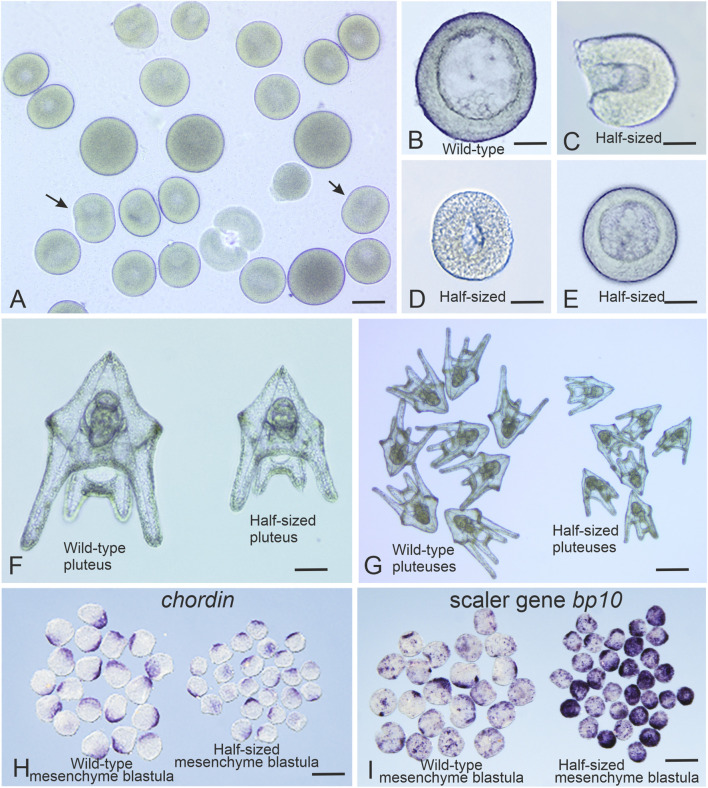
Generation of *Strongylocentrotus droeb*achiensis half-sized embryos. **(A)** Representative outcome of the blastomere separation procedure at the two-cell stage. Arrows indicate dividing single blastomeres. **(B)** Wild-type embryo at the mesenchyme blastula stage. **(C)** Half-sized embryo with an open blastocoel at the early swimming blastula stage. **(D)** Half-sized embryo with a closed blastocoel at the swimming blastula stage. **(E)** Half-sized embryo at the mesenchyme blastula stage. **(F,G)** Full-sized and half-sized pluteus larvae, respectively. **(H)** *In situ* hybridization with a digoxigenin-labeled probe for *chordin* mRNA reveals correct scaling of the *chordin* expression domain in half-sized embryos at the mesenchyme blastula stage. **(I)** *In situ* hybridization with a digoxigenin-labeled probe for *bp10* mRNA shows enhanced expression of this scaler gene in half-sized embryos at the mesenchyme blastula stage. *In situ* hybridization were performed as described in Timoshina et al. (2024). Scale bars: **(A)** 60 μm; **(D,E,G)** 25 μm; **(F)** 30 μm; **(H,I)** 120 μm.

*Note:* For accurate comparisons, unseparated embryos that undergo the full procedure should be used as controls. In high-yield experiments, very few unseparated controls may remain, hence the need for Step 3.

6. Expect a mixture of separated blastomeres, intact two-cell embryos, and occasional unfertilized eggs (Figure 2A).

7. Incubate embryos to the desired stage. The mesenchyme blastula stage is reached at ∼46 hpf at 8 °C and at ∼72 hpf (3 dpf) at 4 °C (Figure 2B).

*Note:* Separated blastomeres initially form open, cup-shaped half-blastulae that may begin swimming (Figure 2C). They typically close a thousand the swimming blastula stage (Figures 2D,E). For later development (e.g., prism or pluteus stages, Figures 2F,G), transfer to deeper culture dishes after swimming onset is recommended.

8. Collect embryos for analysis using a pipette or pipette controller fitted with a wide-bore glass capillary. For bulk collection, a cone of fine mesh (30 μm pore size) can be submerged in a seawater-filled bowl. Pouring embryos into the mesh concentrates them into 2–5 mL for fixation or further processing.

*Note:* Embryos can then be used for studies of embryonic scaling, including *in situ* hybridization (Figures 2H,I).

## 4 Results

### 4.1 Generation of *Xenopus laevis* half-sized embryos through blastomere separation

The development of the protocols described above for generating half-sized embryos from separated blastomeres in *Xenopus laevis* was motivated by two key factors. First, there was a need to produce large numbers of such embryos for studying the phenomenon of embryonic scaling using bulk RNA-sequencing. Second, we encountered significant difficulties when attempting to apply previously published protocols for blastomere separation, including those described by Cooke and Webber (1985a), Cooke and Webber (1985b), and Kageura and Yamana (1983).

**FIGURE 3:**
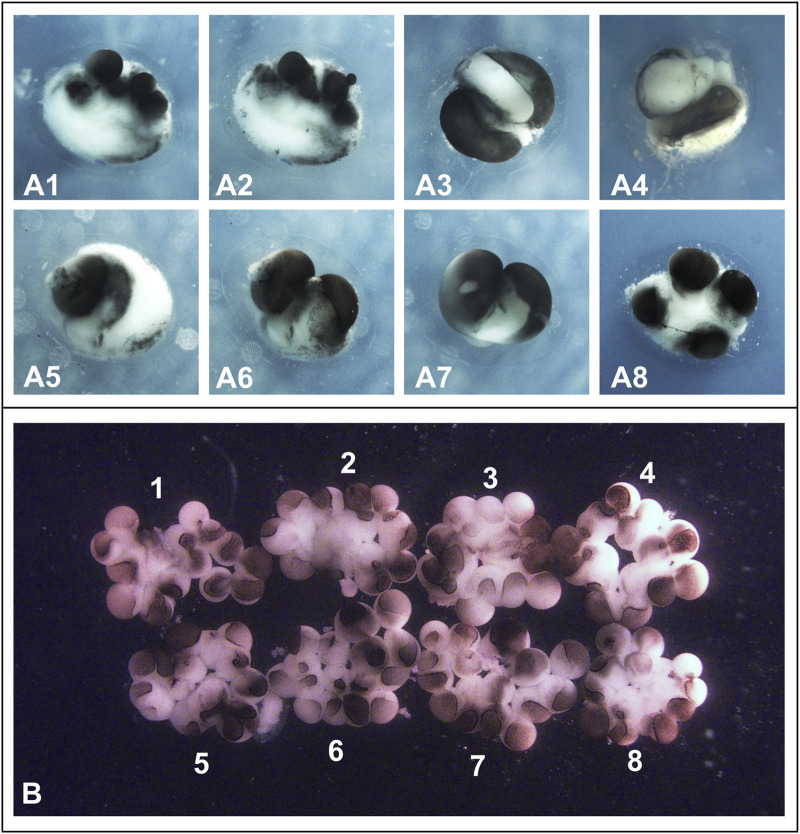
Results of separation and cultivation of *Xenopus laevis* blastomeres according to the protocols described in Cooke and Webber (1985a), Cooke and Webber (1985b), and Kageura and Yamana, 1983. **(A)** Results of blastomere separation in eight 2-cell stage embryos **(A1–A8)** as described in Cooke and Webber (1985a) and Cooke and Webber (1985b), namely by gently blowing the solution from a fine pipette onto the new membrane in the deepening furrows. In all cases, this resulted in either rupturing of the blastomeres or the blastomeres remaining attached to each other if the flow of medium from the pipette was weak. Embryos are shown at 4 cells stage. **(B)** Eight blastomeres obtained by separating embryos at the 2-cell stage were cultured as described by Kageura and Yamana (1983) namely in wells of a tissue culture plate (Corning analog of Falcon-3034) with the bottom covered with 2% agar. Beginning at the two-cell stage, blastomeres gradually lost contact with each other. As a result, by the sixteen-cell stage, embryos 1–8 shown in **(B)** consisted of loosely connected cells and had a flattened shape. Soon after, the blastomeres began to die. Note that the cells appear slightly pink because, following the protocol described by Kageura and Yamana (1983), they were temporarily cultured in 50% Leibovitz (L-15) medium containing phenol red dye.

Specifically, we were unable to reproduce the method reported by Cooke and Webber (1985a), which involves separating blastomeres by directing a stream of medium from a fine pipette across the cleavage furrow. Despite multiple attempts using different batches of eggs from various frogs, all efforts to replicate this technique failed. In every case where we attempted to use this method the blastomeres either remained attached when the flow of medium was weak, or one of them ruptured when the flow intensity was increased. Representative examples of the abnormalities resulting from such attempts are shown in Figure 3A, and corresponding statistics for a triplicate experiment are provided in Table 1.

**TABLE 1:** Number of half-sized embryos developed from blastomeres separated at the two-cell stage and cultured to the mid-neurula stage using different protocols.

Types of embryos and tadpoles	Separation and culturing of blastomeres according to Cooke and Webber (1985a) and Cooke and Webber (1985b)	Separation and culturing of blastomeres according to Kageura and Yamana (1983)	Separation and culturing of blastomeres according to our current protocol
Number of embryos successfully separated at 2-cell stage	0 in total out of 121 (3 independent replicates: 40, 36 and 45 embryos)	82 in total out of 122 (3 independent replicates, 40, 42 and 40 embryos)	80 in total out of 122 (3 independent replicates: 42, 40 and 40 embryos)
Number of half-sized neurula stage embryos successfully developed from separated blastomeres	0	0 in total out of 164	120 in total out of 160
Number of tadpoles that reached stage 46	0	0	60
Number of proportionally scaled tadpoles at stage 46	0	0	14

Embryos for experiments, the results of which are presented in this table, were obtained on different days from three different pairs of frogs. On each day, approximately by 40 embryos at the two-cell stage were separated into blastomeres according to each of the three protocols (a total of approximately 120 embryos on each of the 3 days, for a total of 360 embryos over the 3 days). The data presented in this table represent a single series of experiments performed by two members of our team. However, we consistently obtained the same results across multiple independent trials using different embryo batches, derived from different frog pairs of various ages, and conducted at different times throughout the year.

By contrast, the blastomere separation method described by Kageura and Yamana (1983) proved to be reproducible in our hands. However, when we followed their culturing procedure exactly as described, which involved placing the separated blastomeres into agarose-coated wells of microplates, we consistently observed disintegration of the embryos during cleavage. This led to the formation of amorphous cell aggregates rather than viable embryos. Examples of such outcomes are shown in Figure 3B, and statistical results from a representative triplicate experiment are presented in Table 1. As a result, we were unable to obtain a single viable half-sized embryo using either of these two protocols in their original form.

In contrast to the two previously described protocols, a single experienced researcher can consistently obtain 60–70 pairs of separated blastomeres per experiment using our method. Up to 70% of these develop into morphologically normal pairs of half-sized embryos, although yields depend on the initial quality of the eggs. Approximately 50%–60% of the surviving embryos subsequently reach the swimming tadpole stage, but only about 10% of these tadpoles are well proportioned, appearing as smaller yet otherwise normal versions of their wild-type siblings (Table 1).

### 4.2 Generation of *Strongylocentrotus droebachiensis* half-sized embryos through blastomere separation

The method we describe allows the generation of thousands of half-sized *Strongylocentrotus droebachiensis* embryos with relatively little manual effort. Based on our estimates, this protocol makes it possible to obtain between 3,000 and 8,000 individual blastomeres, or possibly more, separated at the first cleavage from a single *S. droebachiensis* female. This represents a substantial improvement over previously published methods that rely on manual separation with pulled glass needles (Sweet et al., 2004) and over bulk aspiration protocols using glass pipettes (Cameron et al., 1996; Ortiz et al., 2019). Depending on the egg batch, 60%–90% of separated blastomeres developed to at least the gastrula stage. For downstream analyses such as proteomics or RNA-seq, half-sized embryos can be selected at the desired stages using a pipette or a pipette controller fitted with a wide-bore glass capillary. In our experience, one researcher can collect approximately 1,000 half-sized embryos in 2–3 h. This is aided by the relatively slow development of *S. droebachiensis* embryos at 8 °C–10 °C.

Embryos produced using our protocol exhibit robust scaling behavior. This includes proper adjustment of morphogen expression domains, such as *chordin*, as well as compensatory changes in the expression of scaler genes like *bp10*, which regulate morphogen production in a size-dependent manner (Figure 2H) (Timoshina et al., 2024).

Although approximately 10–20 percent of embryos display developmental abnormalities, which are likely caused by mechanical stress during separation, these embryos typically settle at the bottom of the dish and exhibit reduced movement. In contrast, healthy embryos swim actively and rise into the water column, making their identification and selection straightforward.

This procedure can also be applied at the four-cell stage to generate quarter-sized embryos. However, these smaller embryos often fail to scale properly and tend to develop abnormally, consistent with previous observations (Satoh and Kominami, 2008).

## 5 Discussion

In this study, we present optimized protocols for blastomere separation in two widely used model organisms: the African clawed frog (*Xenopus laevis*) and the green sea urchin (*Strongylocentrotus droebachiensis*). While *Xenopus laevis* is a well-established vertebrate model, *Strongylocentrotus droebachiensis* is less commonly used for studying embryogenesis. However, this sea urchin species has one of the broadest geographic distributions among sea urchins and is widely found in the North Atlantic, North Pacific, and Arctic Oceans (Scheibling and Hatcher, 2001).

Based on our observations, *Strongylocentrotus droebachiensis* can be reliably induced to spawn over an extended season using artificial fertilization. We successfully obtained viable embryos from individuals collected in the Barents Sea between February and July. Together, these two species provide complementary platforms for investigating embryonic scaling and developmental regulation across phylogenetically distant lineages.

A distinctive feature of our protocol for producing half-sized *Xenopus laevis* embryos is the combination of two previously published approaches. We use the blastomere separation technique described by Kageura and Yamana (1983), but instead of cultivating the separated blastomeres on an agar surface, as suggested in their paper, we place them into agarose wells that are precisely matched to their size.

The concept of using wells for culturing is mentioned in Cooke and Webber (1985a). However, in that same paper, the authors describe a method for separating blastomeres by directing a stream of medium from a fine pipette into the cleavage furrow. Based on multiple attempts, we found that this method is not applicable for separating blastomeres, at least not at the two-cell stage.

Therefore, only by combining the blastomere separation method of Kageura and Yamana with the culturing approach noted by Cooke and Webber is it possible to create a reliable protocol that consistently yields half-sized embryos from separated blastomeres at the two-cell stage.

While we remain cautious about the feasibility of reliably obtaining half-sized embryos by strictly following the protocols described in the two aforementioned publications, we do not question the validity of the authors’ reported findings on blastomere fate. We have great respect for these established researchers and fully acknowledge the significance of their contributions to the field. It is entirely possible that subtle but important technical details related to blastomere isolation and culture were unintentionally omitted from the published protocols. Additionally, we cannot completely exclude the possibility that, despite repeated efforts by multiple members of our team, we may not have achieved the level of technical proficiency required to successfully perform embryo separation at the two-blastomere stage using the method described by Cooke and Webber (1985a) and Cooke and Webber (1985b).

Our protocol for obtaining half-sized *Xenopus laevis* embryos typically yields dozens of high-quality embryo pairs that are suitable for downstream molecular and phenotypic analyses. However, several technical challenges may arise. One key issue is the variation in egg diameter, both among individual females and within a single clutch, which can complicate the use of uniformly sized agarose wells. Based on our experience, wells made using 1.0 mm capillaries are suitable for most embryos, but 0.7 mm or 1.2 mm capillaries may be necessary to accommodate particularly small or large eggs, respectively. Poorly matched wells can lead to abnormal blastomere spreading and reduced embryo viability.

Second, the success rate depends strongly on egg quality. Eggs from newly introduced females or from individuals that have not been hormonally primed often yield fewer viable embryos. Third, the protocol requires precise timing. The optimal window for blastomere separation occurs shortly after the onset of the first cleavage. Once the animal poles of the forming blastomeres come into contact, mechanical separation without damage becomes difficult or even impossible.

A key innovation of our method for separating the first two blastomeres in sea urchins is the use of gentle extrusion of two-cell embryos through a syringe needle. This approach enables faster and more efficient separation of blastomeres, even in species such as the green sea urchin, where the two blastomeres at the first cleavage stage are tightly adherent.

In sea urchins, blastomere separation can be achieved through mechanical, chemical, or osmotic methods. Among these, chemical and osmotic treatments are less commonly used. For example, *Arbacia punctulata* blastomeres were separated by temporarily increasing the osmolality of seawater (Harvey, 1940). In such cases, twin embryos often remained connected by a thin cytoplasmic bridge and developed in pairs, although the author noted that they eventually became independent.

Johnson et al. (1989) exposed *Lytechinus pictus* zygotes to Evans Blue prior to the first cleavage, which led to blastomere separation in about half of the embryos. However, by the second cleavage, most of the resulting twins remained conjoined.

More commonly, mechanical separation is employed, often in combination with treatment in calcium-free seawater (CFSW). Manual dissection using a pulled glass needle in CFSW has been widely used (Sweet et al., 2004; Suzuki et al., 2025). This method, while effective, requires significant dexterity and patience, and is not well suited for large-scale embryo production.

An alternative approach involves separating blastomeres by aspirating embryos through a pipette in CFSW. This technique yields greater numbers of twin embryos and has been successfully applied to *Strongylocentrotus purpuratus* and *Paracentrotus lividus* (Cameron et al., 1996; Ortiz et al., 2019).

Compared to previously described methods, our technique enables the production of large numbers of half-sized embryos, which is essential for bulk analyses such as Western blotting and various omics approaches. Unlike surgical blastomere separation, it does not require extensive technical skill or time investment.

It is important to note that embryos from different sea urchin species may vary in their developmental and morphological characteristics (as noted in Sweet et al., 2004; Erkenbrack et al., 2019). Consequently, not all protocols are universally applicable. In our case, we used *Strongylocentrotus droebachiensis*, a cold-water species that is seldom employed in molecular studies. Nevertheless, this species introduces its own technical considerations. Being adapted to cold environments, *Strongylocentrotus droebachiensis* requires strict temperature control during embryo handling and culture. Additionally, following blastomere separation, the embryos lack a fertilization envelope, making them particularly fragile during the early cleavage stages.

Before compaction, separated blastomeres are susceptible to fragmentation or aggregation. To minimize these issues, embryos should be distributed sparsely within culture dishes, and the dishes should remain undisturbed until blastula formation. When executed properly, the protocol supports the development of well-scaled half-sized embryos that are suitable for molecular, cellular, and imaging analyses.

In summary, we have developed and described robust and accessible protocols for generating large numbers of half-sized *Xenopus laevis* and green sea urchin (*Strongylocentrotus droebachiensis*) embryos. These protocols are well-suited for studies of embryonic scaling and are compatible with a range of bulk molecular approaches, including transcriptomic, proteomic, and imaging analyses.

## Data Availability

The original contributions presented in the study are included in the article/Supplementary Material, further inquiries can be directed to the corresponding author.

## Supplementary material

SUPPLEMENTARY FIGURE S1 Hand-made equipment for *Xenopus laevis* blastomere separation. **(A)** Glass capillaries of 1.2 mm and 1 mm diameter with sealed tips for making wells for wild-type and half-sized embryos, respectively. Scale bars: **(A)** 1 mm; **(B)** 10 mm.
