# Supplementary figures and images for: Optimized protocols for generating half-sized embryos from separated first two blastomeres in green sea urchin and Xenopus laevis
Source: Front Cell Dev Biol. 2025 Dec 18;13:1730288. doi: 10.3389/fcell.2025.1730288 (PMC12756454; doi:10.3389/fcell.2025.1730288)

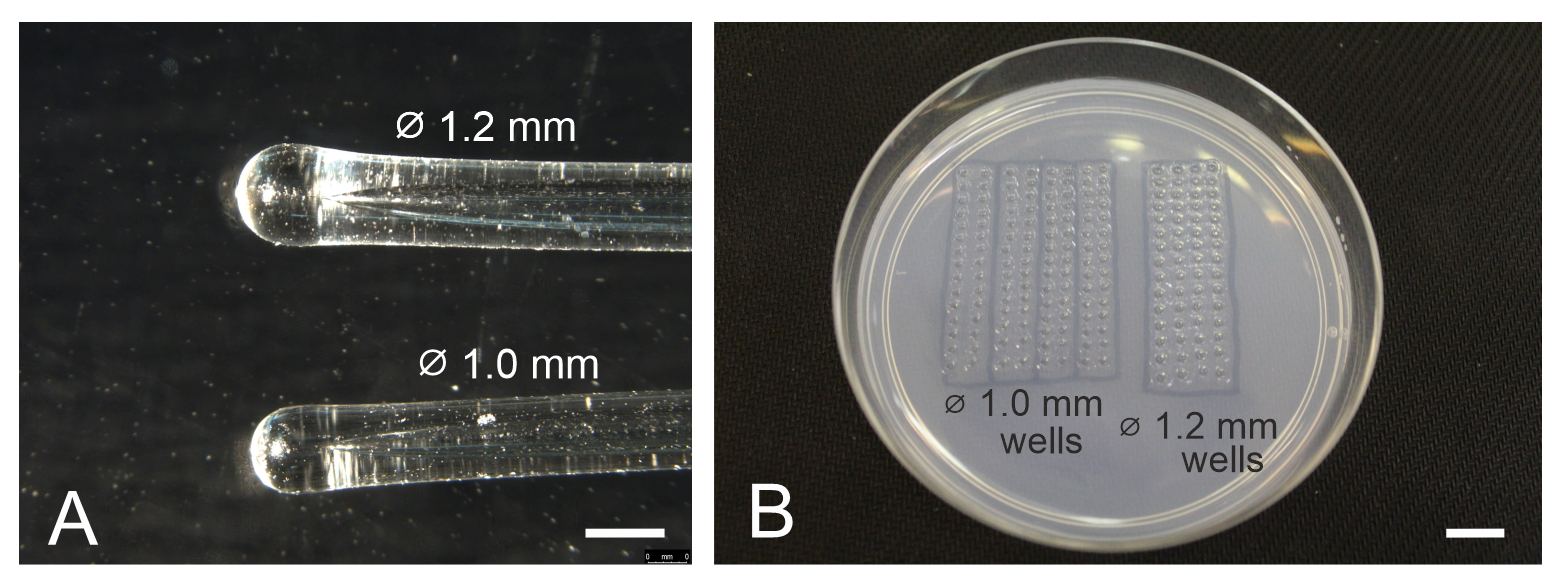

Supplement: Supplementary file 1 [file Image1.tif]
